# Stratification of Patients With Stage IB NSCLC Based on the 8th Edition of the American Joint Committee on Cancer (AJCC) Staging Manual

**DOI:** 10.3389/fonc.2020.00571

**Published:** 2020-04-21

**Authors:** Lei-Lei Wu, Xuan Liu, Wen-Mei Jiang, Wei Huang, Peng Lin, Hao Long, Lan-Jun Zhang, Guo-Wei Ma

**Affiliations:** State Key Laboratory of Oncology in South China, Sun Yat-sen University Cancer Center, Collaborative Innovation Center for Cancer Medicine, Guangzhou, China

**Keywords:** NSCLC, stage IB, prognostic model, survival, treatment strategy

## Abstract

**Objective:** To assess the postoperative prognosis of patients with stage IB non-small cell lung cancer (NSCLC), using a prognostic model (PM).

**Methods:** Patients with stage IB of NSCLC from the two academic databases {the Surveillance, Epidemiology, and End Results [SEER-A, *N* = 1,746 (training cohort)], Sun Yat-sen University Cancer Center [SYSUCC, *N* = 247 (validation cohort)], and SEER-B (*N* = 1,745)} who had undergone lung surgery from 2001 to 2015 were enrolled. The primary clinical endpoint was cancer-specific survival (CSS). Covariate inclusion of prognostic indicators was carried out using a multivariable two-sided *P* < 0.05. We identified and integrated significant prognostic factors for survival in the training cohort to build a model that could be validated in the validation cohort. We used univariate analysis to evaluate the utilized ability of PM in the different races/ethnicities.

**Results:** CSS discrimination in the PM was comparable in both the training and validation cohorts [C index = 0.66(SEER-A), 0.67(SYSUCC), and 0.61(SEER-B), respectively]. Discretization with a fixed PM cutoff of 291.5 determined from the training dataset yielded low- and high-risk subgroups with disparate CSS in the validation cohort (training cohort: hazard ratio [HR] 2.724, 95% confidence intervals [CI], 2.074–3.577; validation cohort: SEER-B HR 1.679, 95% CI, 1.310–2.151, SYSUCC HR 3.649, 95% CI 2.203–6.043, all *P* < 0.05). Our five-factor PM was able to predict CSS; 48-month CSS was 87% in the low-risk subgroup vs. 69% in the high-risk subgroup for the training cohort, while in the validation cohort, they were 80 vs. 73%(SEER-B) and 84 vs. 60% (SYSUCC), respectively. In addition, the results showed that PM with all unadjusted HR > 1 was a significant risk prognostic indictor in white men (*P* < 0.001), Chinese people (*P* < 0.001), and other races (*P* = 0.012).

**Conclusion:** We established and validated a PM that may predict CSS for patients with IB NSCLC in different races/ethnicities, and thus, help clinicians screen subgroups with poor prognosis. In addition, further prospective studies and more cases from different regions are necessary to confirm our findings.

## Introduction

Lung cancer remains the most common cause of cancer-related morbidity and mortality (1). In 2019, in the United States alone, the number of new cases is estimated to reach 228,150, and the death toll is projected to be 142,670 (2). Lung cancer is mainly classified into non-small cell lung cancer (NSCLC) and small cell lung cancer (2). More than 83% of lung cancers are NSCLC (2, 3). According to the 8th edition of the American Joint Committee on Cancer (AJCC) Staging Manual that was implemented in January 2017, the stratification effect on the overall survival (OS) rate is better than that in the 7th edition (4). Owing to the tendency of late diagnosis and tumor recurrence (5), the 5-year OS rate for NSCLC remains low at about 23% (2, 6). The decision of administering adjuvant treatment to patients with stage IB has been controversial. The National Comprehensive Cancer Network guidelines recommend postoperative chemotherapy in patients with high-risk factors, such as vascular invasion, visceral pleural invasion, unknown lymph nodes status, and tumor diameter >4 cm (7); the European Society for Medical Oncology guidelines recommend that adjuvant therapy be given to patients with a tumor diameter >4 cm (8) and the American Society of Clinical Oncology guidelines do not recommend routine treatment for stage IB patients (9). However, following the implementation of the 8th edition, patients with stage IB and tumor size >4 cm have been reassigned to stage IIA (4). According to the 8th AJCC Staging Manual, stage IB is defined by the following: (1) tumor size >3-4 cm, with or without visceral pleural invasion (PL1/PL2); (2) tumor size 0–3 cm, with visceral pleural invasion (PL1/PL2); (3) tumor size 0–3 cm, infringing the main bronchus but with a distance ≥2 cm from the carina or with local pneumonia or with local atelectasis (4).

Many studies have confirmed that tumor size ≥4 cm and visceral pleural invasion can worsen the prognosis of lung cancer patients (10–14).

The 5-year OS of patients with stage IA can be as high as 84%, while the 5-year OS of patients with stage IB is slightly poorer at 68%. With the improvement in early screening for lung cancer, the detection rate of stage I patients increases, and the proportion of patients with stage IB increases (4, 6). Therefore, it is more important to screen for high-risk factors of postoperative poor prognosis in patients with IB as per the 8th edition of the AJCC Staging Manual.

This study used the data of the lung cancer patients recorded in the database of the Surveillance, Epidemiology, and End Results (SEER) and Sun Yat-sen University Cancer Center (SYSUCC) to transform the 8th edition of AJCC Staging Manual based on the information provided. We further analyzed the postoperative prognosis of patients with stage IB NSCLC using a prognostic model (PM) and effectively stratified patients as per the AJCC Staging Manual. We believe that this study will provide important treatment-related information for clinicians and patients.

## Materials and Methods

### Study Cohort

**Figure 1:**
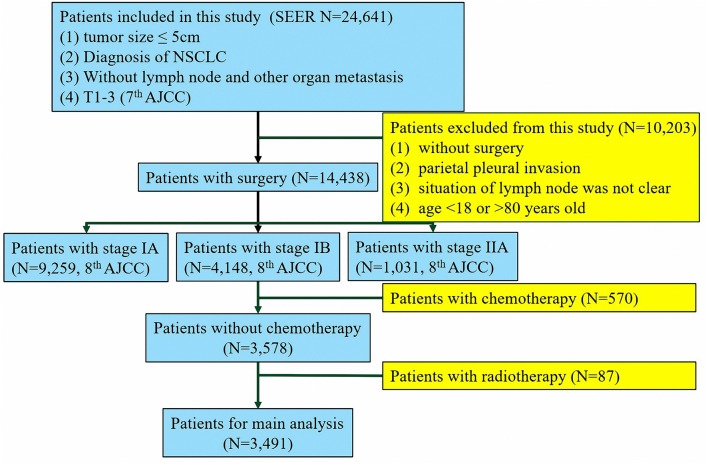
Flow chart of the patient screening process in the the Surveillance, Epidemiology, and End Results.

**Figure 2:**
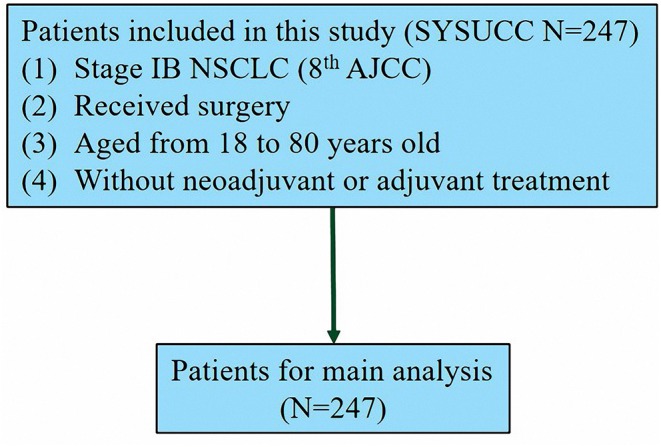
The diagram of the patient screening process in the Sun Yat-sen University Cancer Center.

The study cohort comprised 3,491 patients from the SEER database who underwent lung surgery from January 2010 to December 2015 and 247 patients from SYSUCC who underwent lung surgery from January 2001 to December 2014. Patients who met the following inclusion criteria were enrolled in the study: (1) histopathologic confirmation of NSCLC diagnosis; (2) no distant metastasis to the lymph nodes (LNs) or other organs; (3) pathologically confirmed stage IB as per the 8th edition of the AJCC Staging Manual. Patients were excluded if they (1) had received adjuvant and neoadjuvant cytotoxic chemotherapy or radiotherapy or immune checkpoint inhibitors or underwent other immune therapy regimens; or (2) had a past or current history of another malignancy. According to the patients' records, we translated the pathological staging into the 8th edition of AJCC. The process of patient screening is shown in Figures 1, 2. All patient records were anonymized before analyses. We included information regarding the following patient information: sex, race, age at diagnosis, surgical approach, tumor differentiation, histologic type, number of LNs removed, positive number of LNs, tumor location, tumor extension status, tumor size, pleural invasion (PL), pT stage, pN stage, pM stage, pTNM stage, chemotherapy, and radiation. Patients from the SEER database were randomized into a training cohort (SEER-A) and a validation cohort (SEER-B). SEER-A included 1,746 patients, while the validation cohort included 1,745 patients (SEER-B) and 247 patients (SYSUCC). We obtained approval to use SYSUCC data from the Research Data Deposit of Sun Yat-sen University Cancer Center (Approval number: RDDA2019001261). The primary clinical endpoint was CSS.

### Surgery

According to record in the SEER database and SYSUCC, the main approaches for lung surgery included lobectomy, pneumonectomy, sleeve resection, and sublobectomy (wedge resection and segmental resection). In the SEER database, the average number of LNs removed during surgery was 9.97 ± 0.13, and the median number of LNs was 8.0. However, in the SYSUCC data, the average number of LNs removed during surgery was 20.98 ± 0.79, and the median number of LNs was 19.0

### Histologic Type

Patients exhibited the following histologic types: adenocarcinoma (AC), squamous cell carcinoma (SCC), carcinoid tumor, bronchial alveolar carcinoma (BAC), and neuroendocrine tumor (NT).

### Follow-Up

The survival time and status information was available for these patients. In the SEER database, follow-up duration ranged from 0.0–83.0 months, with an average of 37.0 ± 0.36 months; in the SYSUCC, follow-up duration ranged from 1.0–202.0 months, with an average of 68.6 ± 2.29 months.

### Statistical Analyses

Statistical analyses were performed using SPSS Statistics 25.0 software (IBM SPSS, Inc., Chicago, IL, USA), X-tile software (15), R version 3.5.2 and Graph pad Prism 5. Hazard ratios (HR) with 95% confidence intervals (CIs) were calculated using multivariate regression analysis. Correlations between groups and clinicopathological characteristics were assessed using the χ^2^ test. We then considered information regarding pleural invasion and tumor size and defined patients with both tumor size >3 cm and pleural invasion (TSPI) as TSPI positive, and the other patients as TSPI negative. Multivariate analysis was performed to evaluate the influence of gender, age at diagnosis, race, tumor location, tumor differentiation, surgical approach, histologic type, tumor size and pleura invasion on CSS. A two-sided *p* < 0.05 was considered statistically significant. The most valuable prognostic factors identified using univariate analysis were confirmed with multivariate analysis. Multivariate Cox regression analysis was used to exclude other confounding factors affecting survival. Prognostic indicators were included as covariates in our multivariate analysis with a two-sided *P*-value threshold of <0.05. Similarly, Kaplan–Meier analysis and log-rank tests were used to compare survival curves between groups. Cases were censored when cancer-related death occurred or at the end of follow-up. CSS was selected as the primary clinical endpoint as it was considered the most clinically relevant factor. We adopted a model development and validation approach, using a randomized method to extract the training and validation data sets.

Patient demographics and clinical characteristics were reported for the training cohort. The PM for CSS was constructed by using the linear predictor of the finalized model derived from the training data set. The training cohort was dichotomized into a low-risk and high-risk subgroups using X-tile to determine the cutoff value of PM. A risk score cutoff was defined for classifying patients in the validation cohorts. Concordance C index was generated for discrimination of the multivariable PM.

In the validation cohorts, the PM was applied to calculate the risk score, and patient discretization into the low- and high-risk subgroups was based on the same cutoffs defined in the training datasets.

To investigate the effect of stratification, we screened patients from the SEER database with stage IA and IIA (stages were translated into the 8th edition AJCC), which included 9,259 and 1,031 patients, respectively. We then compared the survival between patients in stage IA, low-risk stage IB, high-risk stage IB, and stage IIA.

## Results

### Patient Characteristics

Clinical characteristics of patients in the SEER database are listed in Table 1. Among the 3,491 patients, 1,630 (46.7%) were men and 1,861 (53.3%) were women; 2,878 (82.4%) were white, 314 (9.0%) were black, and 288 (8.2%) were of other races. Patients' age ranged from 22–80 years (median, 68 years). In this cohort, the 1-, 3-, and 4-year CSS rates were 91.0, 82.0, and 77.0%, respectively, and the median and mean times from surgery to the last censoring date were 34.0 and 37.0 months, respectively. In the training cohort, the 1-, 3-, and 4-year CSS rates were 91.0, 83.0, and 79.0%, respectively, and in the validation cohorts, the 1-, 3-, and 4-year CSS rates were 90.0, 80.0, and 76.0% (SEER-B) and 92.0, 84.0, and 78.0% (SYSUCC), respectively. Clinical characteristics of patients in the SYSUCC are listed in Table 2.

**Table 1:** The associations of clinicopathological characteristics between training cohort (SEER-A) and validation cohort (SEER-B).

	**All patients (*N* = 3,491)**	**Training Cohort (SEER-A, *N* = 1,746)**	**Validation Cohort (SEER-B, *N* = 1,745)**	
**Variables**	**No. of patients (%)**			***P*-value**
Sex				0.446
Male	1,630 (46.7%)	804 (49.3%)	826 (50.7%)	
Female	1,861 (53.3%)	942 (50.6%)	919 (49.4%)	
Age at diagnosis (years)				0.397
≤ 65	1,417 (40.6%)	721 (50.9%)	696 (49.1%)	
>65	2,074 (59.4%)	1,025 (49.4%)	1,049 (50.6%)	
Race				0.745
White	2,878 (82.4%)	1,430 (49.7%)	1,148 (50.3%)	
Black	314 (9.0%)	166 (52.9%)	148 (47.1%)	
Other	288 (8.2%)	144 (50.0%)	144 (50.0%)	
Surgery Approach				0.460
Lobectomy	3,045 (87.2%)	1,521 (50.0%)	1,524 (50.0%)	
Sublobectomy	382 (10.9%)	191 (50.0%)	191 (50.0%)	
Pneumonectomy	62 (1.8%)	34 (54.8%)	28 (45.2%)	
**LNs**				0.285
≤ 8	1,843 (52.8%)	906 (49.2%)	937 (50.8%)	
>8	1,648 (47.2%)	840 (51.0%)	808 (49.0%)	
Tumor grade				0.402
Grade I	616 (17.6%)	321 (52.1%)	295 (47.9%)	
Grade II	1,768 (50.6%)	888 (50.2%)	880 (49.8%)	
Grade III	1,075 (30.8%)	524 (48.7%)	551 (51.3%)	
Grade IV	32 (0.9%)	13 (40.6%)	19 (59.4%)	
Histologic type				0.337^*^
Carcinoid	6 (0.2%)	5 (83.3%)	1 (16.7%)	
BAC	109 (3.1%)	50 (45.9%)	59 (54.1%)	
AC	2,187 (62.6)	1,108 (50.7%)	1,079 (49.3%)	
SCC	895 (25.6%)	443 (49.5%)	452 (50.5%)	
NT	294 (8.4%)	140 (47.6%)	154 (52.4%)	
Pleura invasion				0.412
Negative	1,524 (43.7%)	776 (50.9%)	748 (49.1%)	
Positive	1,696 (48.6%)	839 (49.5%)	857 (50.5%)	
Tumor Location				0.216
Upper lobe	2,114 (60.5%)	1,039 (49.1%)	1,075 (50.9%)	
Middle lobe	210 (6.0%)	117 (55.7%)	93 (44.3%)	
Lower lobe	1,068 (30.6%)	547 (51.2%)	521 (48.8%)	
Other location	84 (2.4%)	38 (45.2%)	46 (54.8%)	

P^*^ value was calculated by Fisher's exact test; P value was calculated by χ^2^ test.

*SEER, the Surveillance, Epidemiology, and End Results; AC, adenocarcinoma; SCC, squamous cell carcinoma; BAC, bronchial alveolar carcinoma; NT, neuroendocrine tumor*.

**Table 2:** The clinicopathological characteristics in Sun Yat-sen University Cancer Center.

**Variables**	**No. of patients (%) *N* = 247**
**Sex**	
Male	153 (61.9%)
Female	94 (38.1%)
**Race/ethnicities**	
Chinese	247 (100.0%)
**Age (years)**	
≤ 65	170 (68.8%)
>65	77 (31.2%)
**Differentiation**	
Grade I	32 (13.0%)
Grade II	127 (51.4%)
Grade III	88 (35.6%)
**Chemotherapy**	
No	247 (100.0%)
Yes	0 (0.0%)
**Radiation**	
No	247 (100.0%)
Yes	0 (0.0%)
**Pleura invasion**	
No	54 (21.9%)
Yes	193 (78.1%)
**Tumor location**	
Upper	133 (53.8%)
Middle	26 (10.5%)
Lower	77 (31.2%)
Other	7 (2.8%)
**Surgery approach**	
Sublobectomy	0 (0.0%)
Lobectomy	242 (98.0%)
Pneumonectomy	5 (2.0%)

In the training cohort, the number of patients who underwent lobectomy was 1,521 (87.1%). Of the remaining patients, 191 (10.9%) and 34 (0.2%) underwent sublobectomy and pneumonectomy, respectively (Table 1). The main histologic type was AC (*N* = 1,180, 67.6%) and SCC (*N* = 443, 25.4%). In this cohort, 839 (48.1%) patients had pleural invasion, with the remaining patients accounting for 51.9% (*N* = 907) of the study population. The majority of tumors were located in the upper lobe (*N* = 1,039, 59.5%), but some were in the lower lobe (*N* = 547, 31.3%), some were in the middle lobe (*N* = 117, 6.7%), and the remaining were in other locations (*N* = 38, 2.2%), including the main bronchi, multiple positions, etc. 906 (51.9%) patients had ≤ 8 LNs removed, while 840 (48.1%) had > 8 LNs removed. Regarding the degree of tumor differentiation, 321 (18.4%) were well-differentiated, 888 (50.6%) were moderately differentiated, 524 (30.0%) were poorly differentiated, and 13 (0.7%) were undifferentiated.

### Univariate and Multivariate Analyses

Univariate and multivariate analyses were performed to investigate the correlations between the clinical characteristics and CSS. As shown in Table 3, univariate analyses identified the following clinical characteristics as significant CSS prognostic factors in patients with NSCLC: gender, age at diagnosis, lobectomy, sublobectomy, LNs, tumor differentiation, AC, SCC, and pleura invasion. Further multivariate analysis based on those characteristics confirmed gender (HR 0.700, 95% CI, 0.542–0.904, *P* = 0.006), age at diagnosis (HR 1.039, 95% CI, 1.023–1.056, *P* < 0.001), LNs (HR 0.974, 95% CI, 0.954–0.994, *P* = 0.012), tumor differentiation (HR 1.496, 95% CI, 1.235–1.813, *P* < 0.001), and pleura invasion (HR 1.459, 95% CI, 1.123–1.894, *P* = 0.005) as independent prognostic factors (Table 3). Our study revealed that these factors were significantly associated with prognosis in stage IB NSCLC patients. Therefore, the five factors mentioned above were useful predictors of postoperative outcome in the training cohort.

**Table 3:** Univariate and multivariate Cox regression analysis for cancer-specific survival in patients with stage IB NSCLC (Cox regression's method is Forward: LR).

	**Univariate analysis**			**Multivariate analysis**		
	**HR**	**95% CI**	***P*-value**	**HR**	**95% CI**	***P*-value**
**Gender**						
Male/Female	0.641	0.502–0.818	**<0.001**	0.700	0.542–0.904	**0.006**
**Age at diagnosis (years)**						
Continuous	1.037	1.022–1.053	**<0.001**	1.039	1.023–1.056	**<0.001**
**Surgery approach**						
Lobectomy	0.655	0.474–0.906	**0.011**	NA	NA	0.084
Sublobectomy	1.457	1.024–2.072	**0.036**	NA	NA	0.371
Pneumonectomy	NA	NA	0.137			
**LNs**						
Continuous	0.973	0.954–0.992	**0.005**	0.974	0.954–0.994	**0.012**
**Tumor differentiation**						
Grade I vs. II vs. III vs. IV	1.486	1.246–1.771	**<0.001**	1.496	1.235–1.813	**<0.001**
**Histologic type**						
Carcinoid	NA	NA	0.067			
BAC	NA	NA	0.464			
AC	0.753	0.589–0.963	**0.024**	NA	NA	0.341
SCC	1.416	1.090–1.840	**0.009**	NA	NA	0.921
Neuroendocrine	NA	NA	0.965			
**Tumor size**						
Continuous	NA	NA	0.062			
**Pleura invasion**						
No/yes	1.547	1.192–2.006	**0.001**	1.459	1.123–1.894	**0.005**
**Tumor location**						
Upper	NA	NA	0.663			
Middle	NA	NA	0.564			
Lower	NA	NA	0.796			
Other	NA	NA	0.891			

*NSCLC, non-small cell lung cancer; AC, adenocarcinoma; SCC, squamous cell carcinoma; BAC, bronchial alveolar carcinoma; NT, neuroendocrine tumor. The meaning of bold values is two-sided P < 0.05*.

### Construction of a PM

**Figure 3:**
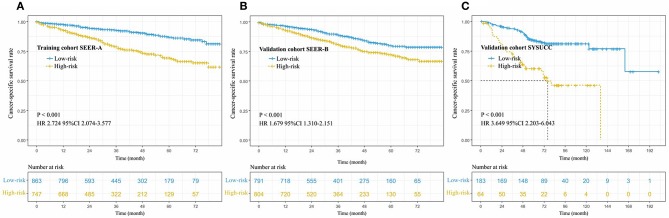
Cancer-specific survival curve for patients with stage IB NSCLC according to the prognostic model in the training cohort **(A)**, internal validation cohort **(B)**, and external validation cohort **(C)**.

Based on the results of the training cohort information analyses, we constructed the PM system and tested the covariates listed in Table 4 for their association with CSS. The PM system was based on weighting (derived from the β-coefficient of the respective log[HRs]) of the five significant covariates in the training cohort (Table 4) that yielded a C index of 0.66 (95% CI, 0.64–0.68) for CSS. This model allowed us to define a high-risk subgroup presenting a significantly reduced likelihood of survival (HR 2.724, 95% CI, 2.074–3.577; *P* < 0.001, Figure 3A). The PM cutoff value was determined in order to distinguish the high-risk group from the low-risk group, using the X-tile software. The cutoff value was 291.5. Our five-factor PM predicted that the 12-month, 36-month, and 48-month CSS in the low-risk subgroup vs. that in the high-risk subgroup was 95.0 vs. 87.0%, 90.0 vs. 74.0%, and 87.0 vs. 69.0%, respectively, in the training cohort, 94.0 vs. 87.0%, 85.0 vs. 75.0%, and 80.0 vs. 73.0% (SEER-B) and 96.0 vs. 81.0%, 91.0 vs. 64.0% and 84.0 vs. 60.0% (SYSUCC), respectively, in the validation cohort.

**Table 4:** Constructed prognostic score to predict cancer-specific survival in stage IB NSCLC patients.

**Covarite**	**β [HR = exp (β)]**	**Score**
Gender	−0.356	−0.356 ^*^ (1/2; male = 1, female = 2)
Age	0.039	0.039 ^*^ Age at diagnosis
Nodes examined	−0.026	−0.026 ^*^ number of nodes examined
Grade	0.403	0.403^*^ (1/2/3/4; Grade I = 1, Grade II = 2, Grade III = 3, Grade IV = 4)
Pleura invasion	0.378	0.378 ^*^ (0/1; no = 0, yes = 1)
		Total computed score ^*^100
**Risk stratification**		
Low risk		≤ 291.5
High risk		>291.5

*NSCLCL, non-small cell lung cancer*.

### Validation of the PM

In order to validate the predictive accuracy of the PM for CSS in IB NSCLC, we tested the PM independently in the validation cohort: an internal cohort of 1,745 patients and an external cohort of 247 patients. The same PM cutoff value of 291.5 allowed us to stratify patients in the validation cohort into the high-risk subgroup with a significantly inferior CSS or the low-risk subgroup (SEER-B: HR 1.679, 95% CI, 1.310–2.151, *P* < 0.001; SYSUCC: HR 3.649, 95% CI 2.203–6.043, *P* < 0.001, Figures 3B,C). The PM in the validation cohorts yielded a C index of 0.61 [95% CI, 0.60–0.63, (SEER-B)] and 0.67 [95% CI, 0.64–0.71, (SYSUCC)] for CSS.

In the SYSUCC, the median survival time of the high-risk subgroup was 76.0 months. However, there was no median survival time in SEER-A, SEER-B, and low-risk subgroup of SYSUCC.

### Effect of Stratification

**Figure 4:**
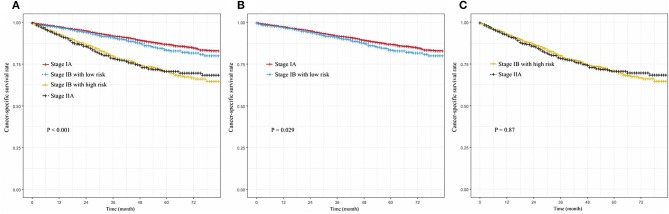
**(A)** Cancer-specific survival curve for NSCLC patients with stage IA, low-risk group of IB, high-risk group of IB, and stage IIA**; (B)** Cancer-specific survival curve for NSCLC patients with stage IA, and low-risk group of IB; **(C)** Cancer-specific survival curve for NSCLC patients with stage IIA, and high-risk group of IB.

To observe the effect of stratification, we screened patients with stage IA and IIA who were translated into the 8th edition AJCC of the SEER database, which included 9,259 and 1,031 patients, respectively. The high-risk and low-risk group stage IB patients were compared with the stage IIA, and IA. We found that stage IA NSCLC patients had the highest CSS in the observation period (*P* < 0.001, Figure 4A). We found that there was no significant difference between stage IA and low risk stage IB in cancer-specific survival (*P* = 0.029, Figure 4B). High-risk stage IB patients did not have a significantly lower CSS than stage IIA patients (*P* = 0.87, Figure 4C).

### Impact of PM on Different Races/Ethnicities

**Figure 5:**
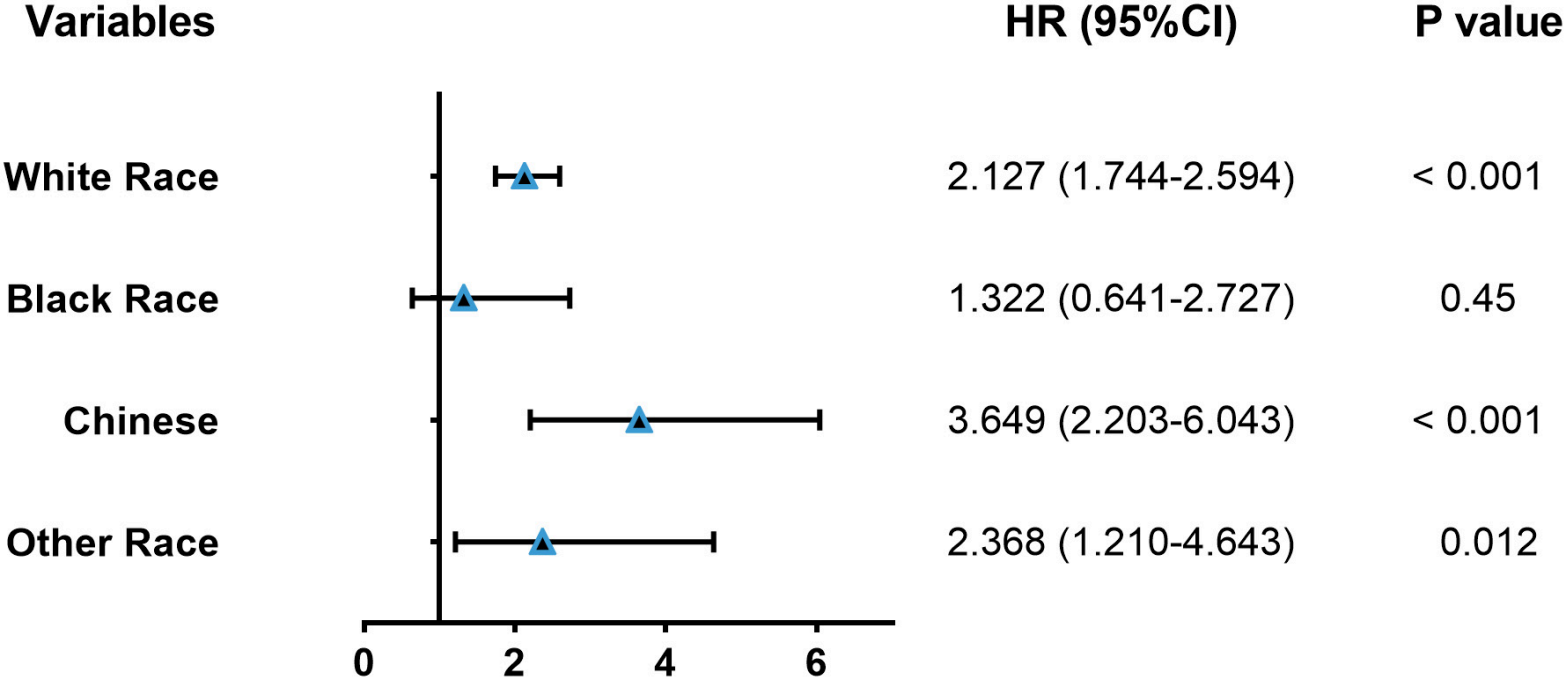
Impact of prognostic model on survival in different races/ethnicities.

We hoped to further explore the impact of PM on different races/ethnicities. Accordingly, univariate analysis was used to estimate the association between PM and CSS. Our results showed that unadjusted HR exceeded 1 or, in other words, PM could be a risk indictor among different races/ethnicities (Figure 5). In addition, there were significant differences in white men (*P* < 0.001), other races (*P* = 0.012), and Chinese people (*P* < 0.001), while no significant differences were observed for black race (*P* = 0.45).

## Discussion

The occurrence and development of NSCLC is complex, and decisions regarding the administration of adjuvant therapy for stage IB NSCLC patients remains controversial. Some research studies have suggested that patients with stage IB NSCLC could benefit from adjuvant therapy (16–18), while other studies have reported no effects of adjuvant chemotherapy on patients (9, 11, 12, 19–21). Studies that have shown the benefit of adjuvant chemotherapy in stage IB patients tend to recommend adjuvant therapy for patients with tumor size ≥4 cm (7, 8, 12, 22, 23). However, stage IB (7th AJCC) disease with a tumor diameter >4 cm has been classified as stage IIA (8th AJCC) (4, 24). One retrospective study based on the 8th edition of the AJCC Staging Manual has shown that postoperative adjuvant treatment could benefit stage IB NSCLC patients (24). A recent meta-analysis, which included 9 randomized collected trials, suggested that patients with stage IB might not need adjuvant chemotherapy; however, the stage IB was based on the 7th AJCC in all trials (25).

**Figure 6:**
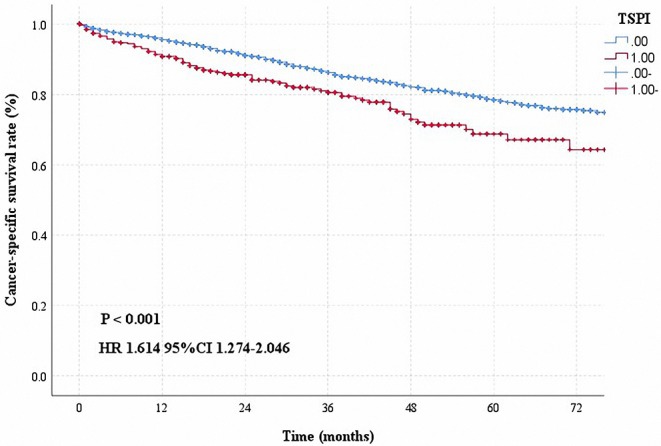
Cancer-specific survival curve for stage IB NSCLC according to the status of TSPI (0: negative, 1: positive).

Based on the above results, some researchers hoped to provide information regarding postoperative treatment decisions by studying the prognosis of early-stage patients. Factors such as age, pathological type, LINE-1 hypomethylation, individualized immune prognostic signature, quality measures, tumor size, preoperative platelet-to-lymphocyte ratio and lymphocyte-to-monocyte ratio, and visceral pleural invasion, were found to influence the prognosis of early-stage patients (7, 10, 11, 13, 14, 22, 26–30). However, the above mentioned studies were unable to individually predict the prognosis of patients. This study aimed to construct an individualized prognostic model and to provide useful information to support clinicians' decisions. We hope to build a simple model by using some commonly obtained patient information. During the course of this research, we analyzed the patients' clinical information, including the indicators shown in Table 1. Eventually, five meaningful indicators were selected using univariate and multivariate analyses of the training cohort, including gender, age at diagnosis, white race, number of nodes removed, tumor differentiation, and pleura invasion. In this study, we considered information regarding pleural invasion and tumor size, based on which we defined patients with both tumor size >3 cm and pleural invasion (TSPI) as TSPI positive, and the rest of the patients as TSPI negative. We found that TSPI could be a risk prognostic factor (Figure 6). During data processing, the number of removed lymph nodes was considered to be a protective prognostic factor (Table 3).

We constructed a PM based on the above five indicators and successfully identified high-risk and low-risk populations in the training and validation cohorts. Our model had a significant impact on patient differentiation (Figure 3), because the C index for predicting CSS rates reached 0.66(SEER-A), 0.61(SEER-B), and 0.67(SYSUCC) in the training and validation cohorts, respectively. Even in comparison with stage IA and IIA, there was no significant difference in survival between the IB stage of the high-risk group and IIA stage (Figure 4). In terms of the clinical application, these indicators can be easily assessed. Information regarding sex and age can be obtained from the admission records, and data on the degree of tumor differentiation, status of pleural invasion, and number of dissected LNs can be obtained from postoperative pathology reports. Clinicians could use the above information and our PM to calculate scores of NSCLC patients with stage IB after surgery, and give patients advice on whether adjuvant therapy is necessary according to prediction of prognosis. In addition, this study included internal and external validation, thus promoting a wide range of applications of the model. According to results of validation of SYSUCC and SEER-B, we found that PM might be applied in different races/ethnicities (Figure 5). We noticed that the clinical popularization of gene test, such as EGFR, in some regions was inadequate (31–33). Therefore, to some extent, this PM in these patients who lack the results of molecular test may have a certain value of utility.

This study has certain limitations. First, the study used the SEER and SYSUCC database in which the distribution of ethnic groups is not balanced. It would be recommendable to include data from different regions in our study, which would balance the race/ethnicity distribution and make the results more generalized. Second, based on the limitations of the SEER database, information on chemotherapy was not comprehensive enough. We do not know whether neoadjuvant chemotherapy or adjuvant chemotherapy was administered, and therefore, when comparing with patients with high-risk stage IB to low-risk stage IB, it is not possible to conclude that patients with adjuvant chemotherapy have a better prognosis. In addition, the number of removed lymph nodes is quite different between SEER and SYSUCC, and the sample size for external validation is small so the number of high-risk patients in the SYSUCC is also relatively small. Thirdly, in the era of precision medicine, molecular detection plays an important role in judging the prognosis and treatment of patients. However, the information of driver genes is incomplete in the data of SEER and SYSUCC. Therefore, based on this research, information on molecular indicators such as EGFR, KRAS, TP53, and ALK can be collected (34–37). Information on these driver genes may increase the predictive ability of PM on CSS. In addition, we couldn't obtain complete information of pathological features such as vascular invasion, which may have an impact on prognosis, in the databases of SEER and SYSUCC. Further, only patients with stage IB NSCLC (8th AJCC) were enrolled; therefore, this model cannot predict or assess CSS in patients with a tumor size ≤ 4 cm and may only be applied to patients with stage IB NSCLC (8th AJCC). Eventually, further prospective and multicenter studies are necessary to confirm our findings.

## Data Availability Statement

Data from this study are available to any interested researchers upon reasonable request to the corresponding author.

## Ethics Statement

The study was approved by the Clinical Research Ethic Committee of SYSUCC (IRB number: B2019-116-01), and informed consent of patients was waived.

## Author Contributions

L-LW designed the research and wrote the article. XL and W-MJ processed data. WH recorded the data of patients. PL, HL, and L-JZ reviewed and edited the article. G-WM analyzed the data and reviewed the article.

## Conflict of Interest

The authors declare that the research was conducted in the absence of any commercial or financial relationships that could be construed as a potential conflict of interest.

## Abbreviations

NSCLC: non-small cell lung cancer
PM: prognostic model
SEER: the Surveillance, Epidemiology, and End Results
SYSUCC: Sun Yat-sen University Cancer Center
CSS: cancer-specific survival
HR: hazard ratio
CI: confidence intervals
AJCC: the American Joint Committee on Cancer
OS: overall survival
PL: pleural invasion
LNs: lymph nodes
AC: adenocarcinoma
SCC: squamous cell carcinoma
BAC: bronchial alveolar carcinoma
NT: neuroendocrine tumor
TSPI: both tumor size of > 3 cm and pleural invasion.
